# The Relationship Between Dietary Sodium Intake and Cognitive Function: A Narrative Review

**DOI:** 10.1007/s13668-026-00750-8

**Published:** 2026-03-21

**Authors:** Samantha L Gardener, M Fu, L Morini, T Schwartzkopff, G Savage, RN Martins, SR Rainey-Smith

**Affiliations:** 1 Centre of Excellence for Alzheimer’s disease Research and Care, School of Medical and Health Sciences, Edith Cowan University, 270 Joondalup Drive, Joondalup, WA 6027 Australia; 2 Alzheimer’s Research Australia, Sarich Neuroscience Research Institute, Perth, WA Australia; 3 School of Psychological Sciences, Macquarie University, North Ryde, Sydney, NSW Australia; 4 Department of Biomedical Sciences, Macquarie University, Camperdown, Sydney, NSW Australia; 5 Centre for Healthy Ageing, Health Futures Institute, Murdoch University, Murdoch, WA Australia; 6 School of Psychological Science, University of Western Australia, Perth, WA Australia

**Keywords:** Alzheimer’s disease, Sodium, Salt, Ccognition, Hypertension

## Abstract

**Purpose of Review:**

This narrative review provides discussion on current evidence regarding the relationship between sodium intake and cognitive function in animal and human studies, as well as potential mechanisms underlying this relationship.

**Recent Findings:**

Recent evidence suggests high sodium intake is associated with development of cognitive impairment and neural dysfunction. Additionally, studies have proposed that high sodium intake is associated with increased aggregation of Aβ-amyloid and that hypertension (for which high sodium intake is a risk factor) modulates the relationship between Aβ-amyloid and development of cognitive impairment and Alzheimer’s disease. However, while animal studies demonstrate a consistent relationship between high sodium intake and cognitive impairment, this relationship remains less clear in humans.

**Summary:**

Overall, mixed results were observed regarding whether sodium intake is associated with cognitive function. To a certain extent, the findings from this review support the notion that high sodium intake could be having a negative impact on middle and older-aged individuals’ cognitive health. Further exploration of the relationship between dietary sodium intake and cognition is needed in well characterised human cohorts, using comprehensive assessment of cognitive function. Furthermore, given self-report sodium intake can give over- or under-reported levels, the addition of 24-hour urinary sodium levels would enhance research findings and its interpretation.

**Supplementary Information:**

The online version contains supplementary material available at 10.1007/s13668-026-00750-8.

## Introduction

Alzheimer’s disease (AD) and its dementia syndrome presents one of the most prominent healthcare challenges in the modern era, bringing with it widespread social, economic, and personal burdens. The World Health Organisation (WHO) estimated the prevalence of dementia cases in 2021 to be around 57 million globally with the total number of individuals living with dementia expected to reach 153 million in 2050 and the economic burden of dementia estimated at US$1.3 trillion in 2019 [1]. Moreover, dementia affects the most disadvantaged and vulnerable groups in society with lower socioeconomic status associated with both higher incidence and mortality [2], and with over 50% of people in aged care facilities affected [3]. Dementia is also associated with great challenges for family members and carers, who are at increased risk for developing depression [4].

However, currently no cure for dementia exists, thus the development of preventative strategies are essential in delaying disease onset and/or slowing disease progression. In particular, recent evidence has suggested that a Mediterranean style dietary pattern is associated with better executive function performance in cognitively normal older adults [5], and importantly, has been shown to be associated with decreased accumulation in the brain of Aβ-amyloid, a pathological hallmark of AD [6].

Dietary recommendations for dementia prevention need to be based on strong scientific and clinical evidence and currently it remains unclear as to whether specific components of these dietary patterns (such as low salt or low fat) are more crucial than others in terms of their neuroprotective properties. Recent evidence has suggested an association between high salt intake and the development of cognitive impairment and neural dysfunction [7, 8]. In particular, rodent studies have demonstrated that a high salt diet was associated with impaired performance on hippocampal-dependent spatial memory tasks including the Morris water maze [9], novel place recognition task, and fear conditioning tasks measuring long-term memory retention [10].

Evidence of an association between high dietary salt intake and cognitive impairment in humans, however, appears less consistent [11–13]. The mechanisms underlying the association between a high salt diet and cognitive impairment also remain unclear, with few animal studies investigating the role of hypertension [14], whilst no studies to date have investigated brain tau (another AD hallmark neuropathological feature) or Aβ-amyloid in relation to salt intake in humans.

The literature review that follows will include more detailed discussion of the current evidence of the relationship between high sodium diet and cognitive function in animal and human studies, as well as potential mechanisms underlying this relationship.

## Methods

A computer-based search of PubMed was conducted for published articles using sodium or salt in the search terms, with cognitive function, magnetic resonance imaging (MRI) variables, or pathological cerebral hallmarks of AD (Aβ-amyloid or tau) as the primary or secondary outcome. The search terms are listed in Supplementary Table 1. The search was limited to articles in humans or animal models and published in English.

**Table 1:** Dietary intervention animal studies published investigating sodium consumption and cognitive outcomes

Title, Reference	Cohort Information	Study Design	Sodium excreted/ingested	Outcome Measure(s)	Main Finding(s)
High Salt Elicits Brain Inflammation and Cognitive Dysfunction, Accompanied by Alterations in the Gut Microbiota and Decreased SCFA Production	Adult Female C57BL6 mice Age: 8–10 weeks Wt: 20–24 g	8-week intervention study	Normal chow (control); 8% NaCl (HSD)	MWM, DNA extraction from feces and microbiota analysis, Fecal SCFAs analysis, Blood brain barrier permeability	Special learning and memory abilities of the HSD group were significantly damaged - mice spent more time trying to find the platform than those in the control group, with the number of platform crossings also significantly reduced. HSD induced blood brain barrier dysfunction and microglial activation in the brain, an inflammatory environment with increased expression levels of IL-1β, IL-6, and TNF-α in the cortex
Dietary salt promotes neurovascular and cognitive dysfunction through a gut-initiated TH17 response	Age: 8 weeks Male C57BL6 mice	4-to-24-week intervention study	Normal chow (control); 4% or 8% NaCl (HSD)	Novel object recognition test, Barnes maze test, resting cerebral blood flow, vascular inflammatory enzymes,	Mice on 4% NaCl showed impairment after 12 weeks, while those on 8% NaCl showed impairment at 8 weeks.
A High-Salt Diet Further Impairs Age-Associated Declines in Cognitive, Behavioral, and Cardiovascular Functions in Male Fischer Brown Norway Rats	Male Fisher Brown Norway Rats Age: Adult (2 months), Old (20 months)	4-week intervention study	0.4% NaCl (control); 8% NaCl (HSD)	Open-field and light-dark anxiety behavior test; Radial arm water maze learning and memory function test; Brain dissection and analysis.	HSD resulted in more anxiety-like behavior in older rats, and impaired results from a radial arm maze test, compared to adult HSD, adult normal salt, and older normal salt rats.
High salt diet impairs memory-related synaptic plasticity via increased oxidative stress and suppressed synaptic protein expression	Male C57BL/6J mice Age: 6–8 weeks Wt: 18–22 g	4-to-7-week intervention study	0.4% NaCl (control); 8% NaCl (HSD)	Object-place recognition task; Fear conditioning memory test; open-field anxiety test	No difference in physical condition or anxiety measures in HSD compared to normal salt diet. HSD def mice showed poorer performance in the fear conditioning test and had greater disturbances in hippocampal LTP, greater hippocampal ROS and down regulation of the expression of synapsin 1, synaptophysin, and BDNF.
High salt induced hypertension leads to cognitive defect	Male Sprague-Dawley rats Age: 2 months Wt: 250 ± 20 g	9-week intervention study	0.26% NaCl (control); 8% NaCl (HSD)	Non-invasive blood pressure, Cerebral blood flow measurement, Open field activity test, Fear conditioning (hippocampus-dependent associative memory), MWM, Golgi staining, Nissl staining, Intracellular calcium concentration, Western blotting, ELISA quantification of Aβ.	HSD significantly raised systolic blood pressure. HSD fed rats displayed similar anxiety to control animals. MWM test showed no difference between groups in finding the hidden platform, however, time to locate the target quadrate in the HSD group confirmed impairment in spatial memory retrieval, indicating hippocampus-dependent memory deficits. There was no significant difference between groups for tau phosphorylation.
High-salt diet enhances hippocampal oxidative stress and cognitive impairment in mice	Male C57BL/6J Mice Age: 6–8 weeks old Wt: 18–22 g	12-week intervention study	0.4% NaCl (control); 7% NaCl (HSD)	MWM; Blood pressure; heart rate; blood glucose; determination of superoxide anion; measurement of SOD, CAT, GSH and T-AOC	No significant difference in blood pressure, body weight, heart rate or blood glucose. Spatial learning and memory via MWM, no significant differences. Spatial memory via probe trial, HSD group had significantly decreased time and frequency in targeted quadrant. HSD group exhibited a higher level of hippocampal superoxide, while significantly reduced levels of GSH and T-AOC, and reduced SOD and CAT activity.
Dietary Salt Disrupts Tricarboxylic Acid Cycle and Induces Tau Hyperphosphorylation and Synapse Dysfunction during Aging	CB7BL/6 Mice N2A cells	3-months study	0.5% NaCl (control): 8% NaCl (HSD) NCM: 40mM (NaCl medium)	MWM: TCA metabolites and enzymes measured with liquid chromatography-tandem mass spectrometry, western blotting, immunofluorescence.	Elevated salt intake impairs the TCA cycle (expression of metabolites associated with glucose metabolism) and induces tau hyperphosphorylation and synapses dysfunction during aging, which ultimate result in cognitive impairment.
High salt induces cognitive impairment via the interaction of angiotensin II-AT1 and prostaglandin E2-EP1 systems	Male C57BL/6 N Mice	12-weeks study	Normal water (control): 2% NaCl (HSD)	Social interaction test: Novel object recognition test: Open field, Y-maze and Barnes-maze paradigms.	Social behavior is more sensitive to HS intake than recognition memory.

*Abbreviations*: *BDNF* brain derived neurotrophic factor, *CAT* catalase, *DNA* deoxyribonucleic acid, *GSH* Glutathione, *HSD* high salt diet, *IL-1β* interleukin-1β, *IL-6* interleukin-6, *IL-17* interleukin-17, *LTP* long-term potentiation, *MWM* Morris Water Maze, *NaCl* Sodium Chloride, *NO* nitric oxide, *ROS* reactive oxygen species, *SCFAs* Short-chain fatty acids, *SOD* superoxide dismutase, T-AOC Total Antioxidant Capacity Colorimetric, *TH17* T helper 17 cell, *TNF-α* tumour necrosis factor-alpha, *Wt* weight

Titles and abstracts were screened by the first author to ensure they examined the relationship between sodium or salt intake and one of the outcome measures. Articles were included if they reported prospective, retrospective or intervention studies and were either cross-sectional or longitudinal in nature. There were no strict criteria relating to the inclusion of controls or the quality of these controls with respect to matching for other macro or micronutrients. Studies were excluded if they were: (1) not peer-reviewed, (2) a conference proceeding abstract, (3) a review or theoretical article, (4) published in a language other than English, or (5) conducted in children (age < 18 years). The reference lists of included articles were screened and additional articles which met inclusion criteria were added via hand search.

### Sodium Intake and Cognitive Function in Animal Studies

An increasing body of preclinical evidence indicates that excessive dietary sodium intake is associated with cognitive impairments, particularly affecting spatial and long-term memory. Experimental models, predominantly involving rodents, have demonstrated consistent neurocognitive deficits following chronic consumption of high salt diets, commonly defined as those containing ≥ 4% sodium chloride (NaCl).

Several studies utilizing C57BL/6 mice have shown that elevated salt intake impairs spatial memory. For instance, female mice maintained on an 8% NaCl diet for 8 weeks exhibited significant deficits in spatial learning in the Morris water maze test compared to a control group. These behavioural impairments were accompanied by blood-brain barrier (BBB) disruption, microglial activation, and a pro-inflammatory profile in the cortex, including upregulation of interleukin-1β (IL-1β), interleukin-6 (IL-6), and tumour necrosis factor-alpha (TNF-α). Furthermore, increased neuronal apoptosis was observed in both the cortex and hippocampus [15]. In male C57BL/6J mice, exposure to a high salt diet for seven weeks resulted in spatial memory impairments in the novel object-place recognition task compared to control mice fed 0.4% NaCl, while a four-week exposure was sufficient to impair long-term memory in a fear conditioning test. These findings suggest a time-dependent effect of high salt consumption on cognitive function. The observed impairments were associated with elevated oxidative stress, downregulation of synaptic proteins (e.g. synapsin 1, synaptophysin), and reduced brain derived neurotrophic factor expression in the hippocampus [10].

Liu et al. [9] further demonstrated that a 12-week high salt diet (7% NaCl) in male C57BL/6J mice impaired spatial memory retention compared to mice fed a normal salt diet (0.4% NaCl), as indicated by poorer performance in the probe test of the Morris water maze. Spatial learning and memory were not significantly different between high salt fed mice and normal salt fed mice, nor was swimming velocity suggesting impaired retention was not due to altered motor ability. This deficit correlated with decreased antioxidant defence capacities in the hippocampus (but not the cerebral cortex) implicating oxidative stress in memory impairment.

Similar outcomes have been reported in rat models, Guo et al. [14] found male Sprague-Dawley rats fed an 8% NaCl diet for nine weeks exhibited impaired spatial memory retrieval in the probe trials of the Morris water maze and deficits in contextual fear conditioning (long-term memory of a fearful context) compared to standard low salt fed rats (0.26% NaCl). As with Lui et al.’s results, swimming speed remained comparable confirming motor function was not affected. These cognitive effects coincided with elevated systolic blood pressure inducing a chronic and sustained hypertension linked to a decrease of cerebral blood flow, resulting in a loss of dendritic spines in the hippocampus and a downregulation of the memory related CaMKII/CREB signalling pathway. Notably, tau phosphorylation and Aβ-amyloid levels remained unchanged, suggesting a non-AD-specific mechanism underlying the cognitive decline.

The influence of age on vulnerability to high salt-induced cognitive dysfunction has also been examined. In a study by Chugh et al. [16], a four-week 8% NaCl diet impaired short-term memory on the radial arm maze in 20-month-old male Fischer brown Norway rats, while younger (2-month-old) adults and low salt diet fed control rats (0.4% NaCl) remained unaffected. Oxidative stress variables were increased, and the antioxidant enzyme glyoxalase-1 expression in the hippocampus and amygdala reduced in the old rats compared to the adult rats and both control groups, however other antioxidant enzymes remained unchanged. Interestingly, the old rats fed a high salt diet al.so exhibited increased anxiety in an open field test while this was not the case for adult rats, which suggests that a high salt diet may potentially increase the hypothalamic–pituitary–adrenal (HPA) stress response in older animals.

The effects of both dietary salt concentration and exposure duration were further highlighted in a study investigating nonspatial memory performance in mice aged 2 and 12 months. High salt diets containing 4% and 8% NaCl impaired performance in the novel object recognition task, with deficits emerging after 12 weeks and 8 weeks, respectively, demonstrating a dose–response relationship and suggesting that even a lower concentration of sodium than that typically used in prior studies was sufficient to induce impairment, given enough time. Spatial memory deficits were also observed in mice fed the high salt diet in the Barnes maze, a hippocampus-dependent task [17].

Kubota et al. [18] reported that male mice on a 2% NaCl diet for 4 to 12 weeks displayed impairments in social behaviour and object recognition memory compared to the control group, accompanied by tau hyperphosphorylation, down-regulation in synapse-related proteins, and dendritic degeneration in the pre-frontal cortex and hippocampus. Treatment with a BBB-crossing AT1 receptor blocker mitigated these effects, implicating the involvement of both the angiotensin II type 1 (AT1) and prostaglandin E2 (PGE2)-EP receptor systems in salt-induced neuropathology.

Yuan et al. [19] investigated the specific effects of a high salt diet on synapses and tau hyperphosphorylation in parallel on aged male C57BL/6 mice and N2a cells grown in NaCl medium (containing an additional 40mM NaCl). They observed synaptic loss, tau hyperphosphorylation, and subsequent cognitive decline, linking for the first time to impairments in the tricarboxylic acid (TCA) cycle. Elevated glucose metabolism-related metabolites suggested metabolic disruption as a contributing factor to tau hyperphosphorylation and synaptic dysfunction.

While animal studies have demonstrated a consistent association between high salt intake and poor cognitive function with age and exposure duration acting as critical modulators, it is important to note that the concentration of sodium in that of high salt diet animals is often 15–20 times greater than that in control animals [7]. Thus, it is argued that this is not a valid representation of human models, whereby the maximum amount of salt consumed in a high salt diet is generally only double the normal amount.

Table 1 describes dietary intervention animal studies published investigating sodium consumption and cognitive outcomes.

## Sodium Intake and Cognitive Function in Human Studies

While animal studies demonstrate a consistent association between high sodium intake and poor cognitive function, studies of humans have produced inconsistent results. A six-month randomised controlled trial evaluating the effects of aerobic exercise and diet in older-aged sedentary adults (age > 55 years) found that reducing sodium intake over the trial duration was associated with improved executive function but not memory or language/verbal fluency [20]. Whilst participants were excluded if they had a dementia diagnosis, they were required to have subjective memory complaints, objective evidence of cognitive impairment, and at least one cardiovascular disease (CVD) risk factor. The authors did note that the assessment of language/verbal fluency was limited to only Controlled Oral Word Association Test and Animal Naming tests, which may not have been adequate to assess this domain. Furthermore, sodium intake was estimated using a four-day food diary. Although prospective recording can minimise recall bias, this method may prompt participants to alter their usual eating behaviours because they are aware their intake is being monitored. In addition, a four-day recording period may not adequately reflect longer-term dietary patterns or seasonal variations, potentially limiting its ability to represent habitual intake accurately.

A prospective follow-up study of 6,426 women (aged 65–69) with a median follow-up of 9.1 years found that higher baseline self-reported sodium intake (> 1500 mg/day) was not associated with increased risk of mild cognitive impairment (MCI) or probable dementia as assessed by the Modified Mini-Mental State Examination (3MS) and further neurocognitive and neuropsychiatric examinations. However, while formal interactions were not significant, there was an overall trend to suggest that women with hypertension or those receiving antihypertensive medication reporting higher sodium consumption were at greater risk of developing cognitive decline [12]. Sodium intake was calculated from a food frequency questionnaire (FFQ), however separate analyses based on 24-hour urine excretions in a subset of women were conducted to correct dietary self-report data of sodium intake for potential measurement errors, although this did not result in any changes to the observed findings. After applying calibration equations based on 24-hour urine excretions method, the exposure distribution of the study sample changed reflecting higher actual sodium consumption than as assessed by FFQs.

A longitudinal study of 1,262 older adults (aged 67–84) found that amongst those with low levels of physical activity, a sodium diet (calculated using an FFQ) in the lowest tertile was associated with reduced decline in cognitive performance on the 3MS over a three-year period compared to those who had a high or mid-level sodium diet. This effect, however, did not hold for those who engaged in high levels of physical activity (i.e., there was no significant difference in levels of cognitive decline across sodium intake tertiles), suggesting that a low sodium diet had a protective effect for sedentary individuals only. Interestingly, the degree of cognitive decline across sodium tertiles for those in the high activity group was generally less than the decline seen in the low activity group. This suggests that physical activity may have an overall protective effect on the vasculature and cerebral system which attenuates the damage caused by a high salt diet [21]. Indeed, the link between physical activity and cognition has been shown in previous studies and a meta-analysis of randomised controlled trials showed that regular moderate exercise improved cognitive function in older adults with and without existing cognitive impairment [22].

Intriguingly, Rush et al.’s [13] cross-sectional study of 925 individuals (mean age 74.5 ± 8.7) found that those placed in the lowest sodium intake quartile performed worse on a cognitive switching task and had increased odds of clinically significant cognitive impairment on the Mini-Mental State Examination (MMSE) compared to those with higher sodium intake, with no associations observed on the Verbal Fluency Test, independent of lifestyle factors, comorbidities, medication use and kidney function. While this finding appears to contradict the general literature, it may be explained if individuals in the very low sodium group had needed to adopt a low sodium diet due to pre-existing health conditions that affect cognitive function. However, while individuals in the lowest sodium quartile had higher rates of hypertension compared to the middle two quartiles, controlling for a variety of health-related variables, including hypertension, did not change the results. It is possible that lowering dietary sodium may adversely impact insulin regulation as well as the renin-angiotensin and sympathetic systems and this may adversely affect cognitive function [23–25].

Furthermore, in women, “sometimes” adding salt to food after cooking was found to be associated with better cognitive function than women who “never” added salt after cooking (β = 0.98, 95% CI 0.25, 1.71). Whilst in men, “usually” adding salt to food during cooking was associated with poorer cognitive function than men who “never” added salt [β = -1.37, 95% CI -2.39, -0.35; 26]. In this study, dietary data were collected in 2010 and 2014 using an FFQ, and cognitive function was measured in 2014 using the Telephone Interview of Cognitive Status. The association observed in woman used the 2010 diet data, and the association observed in men used the 2014 diet data. Participants were aged 55–64 years and resided in Victoria, Australia (*n* = 617). Although high sodium intake levels are considered to be damaging to health, sodium is an essential nutrient and low intakes in older adults have been associated with mortality [27].

Klinedinst et al.’s [28] prospective study of 1,787 middle- to older-aged adults (aged 46 to 77 years at completion of the study) found that “usually” adding salt to meals was associated with worse fluid intelligence test (FIT) scores over time, but only for those with genetic risk factors including a familial history of AD (mother or father) and presence of the apolipoprotein E (APOE) ε4 allele. The FIT score is quantified by how many numeric, logic, and syntactic questions (out of 13 total questions) that participants were able to answer correctly within two minutes. The FIT test was administered at baseline (2006–2010) and two follow-ups (2012–2013, and 2015–2016).

Another longitudinal study of 1,194 older adults (mean age 74 ± 3 years) found that higher sodium intake at baseline was not predictive of greater risk of cognitive decline over a follow-up period of 6.9 ± 0.1 years as measured by change in the 3MS, nor was it associated with micro- or macro-structural brain changes measured by MRI in a subset of 248 participants [29]. The measure of cognitive function was limited in this study, with only the 3MS administered.

In an Australian cross-sectional study of 48 participants with no serious cognitive impairment (mean age 78.2 ± 61 years), sodium intake was not significantly correlated with a range of cognitive measures from the Verbal Fluency Test, Rey Auditory Verbal Learning Test, Boston Naming Test, Digit Span Backwards test, and Trail-Making Test [30]. However, this was a small cohort with an under-representation of people with lower education levels, and sodium intake was calculated from a limited three-day food diary. A cross-sectional study of 44 Irish participants (mean age 57.7 ± 9.4 years) at risk of dementia due to having a first degree relative with AD found that sodium intake was significantly higher in those participants with a low MMSE (between 24 and 17). Of the 44 participants, however, only 4 had a low MMSE [31].

While food frequency questionnaires (FFQs) are accepted as generally providing an accurate estimation of an individual’s sodium intake, particularly over a long period of time, methods that do not rely on self-report may provide additional insight. One such study assessed sodium intake using 24-hour urine sodium excretion and found that in 119 adults newly diagnosed with hypertension (mean age 54.2 ± 16.1), higher levels of sodium excretion (an indicator of higher dietary sodium intake) were associated with worse scores on the Standardised MMSE (SMMSE) [11]. This study was cross-sectional in nature so a causal relationship cannot be inferred. Furthermore, the study used a single urine collection and daily variability can be observed in urinary sodium excretion of individuals; as such, interpretation of findings is limited.

In a recent cross-sectional Chinese study of 561 community-based individuals (aged 52.7 ± 9.3 years), 24-hour urinary sodium to potassium ratios were negatively associated with MMSE scores, and with 24-hour urinary sodium to potassium ratios divided into tertiles, the middle and highest tertiles showed 2.01-fold (95% CI 1.03, 3.93; *p* = .041) and 3.38-fold (95% CI 1.77, 6.44; *p* ≤ .001) higher odds for presence of MCI compared with the lowest tertile. MCI diagnosis was defined only using an MMSE score, and urinary sample was only collected once, which again, wouldn’t show daily or seasonal variability in urinary sodium excretion; both features of the study can be considered limitations [32].

Recent studies attempted to strengthen the hypothesis of a relationship between sodium and potassium dietary intake and cognitive impairment, and to investigate the physiological mechanism underpinning this correlation. A prospective Chinese study conducted in a cohort of 4213 participants (aged > 50 years at baseline) collected dietary data using the 24-hour dietary recall method, and participants’ objective and subjective cognitive functions were assessed with the Telephone Interview for Cognitive Status-modified (TICS) [33]. Results showed that higher average sodium and sodium/potassium intake, and lower potassium intake had a negative impact on cognitive functions, but similar to other studies epidemiological evidence is not strong, even in a Chinese population where daily sodium intake is significantly higher than the global average. The associations of dietary potassium and sodium/potassium with cognitive decline were indirectly mediated by cardiovascular and cerebrovascular disease, as well as sleep time, such that salt could reduce sleep time, and sleep time was positively associated with impaired cognitive function.

Detrimental effects of dietary salt on the cardiovascular system may lead to cognitive damage. This hypothesis has been further investigated by Liu et al. [34]: 2041 participants (aged 68.51 ± 6.13) underwent a Global Cognitive Function Assessment (including the MMSE, Montreal Cognitive Assessment [MoCA], and Dementia Rating Scale [DRS]) and a 24-hour urine collection for salt intake estimation. Participants were monitored for 11.4 ± 2.0 years. Decreases in global cognitive function differed significantly among groups even after statistical adjustments, and in particular, decline was greater in the high salt intake group; thus, it was strongly associated with an accelerated decline in global cognition, independently of known risk factors.

The InCHIANTI epidemiological study collected data from 1270 participants aged 73.5 ± 8.8. Twenty-four-hour sodium urinary excretion and 24-hour nitrate urinary excretion, together with dietary sodium and nitrate (calculated from an FFQ) showed no association with cognitive performance test scores (MMSE, Trail Making Test A and B), whilst only high nitrate levels were related to lower blood pressure values independent of dietary sodium intake [35].

Table 2 describes dietary intervention human studies published investigating sodium consumption and cognitive outcomes.

**Table 2:** Dietary human studies published investigating sodium consumption and cognitive outcomes

Title, Reference	Cohort Information	Study Design	Sodium intake/excretion	Outcome Measure(s)	Main Finding(s)
The Relationship Between Cognitive Function, Depressive Behaviour and Sleep Quality with 24-h Urinary Sodium Excretion in Patients with Essential Hypertension	*N* = 119 Mean age: 54.2 ± 16.1 Konya, Turkey	Cross-sectional study	Sodium 24-h excretion; mean 140.4 ± 3.4 mmol/L	SMMSE	Higher sodium excretion associated with worse SSMSE.
Dietary Sodium/Potassium Intake Does Not Affect Cognitive Function or Brain Imaging Indices	*N* = 1194 Mean age: 74.1 ± 2.8 Pittsburgh, PA and Memphis, TN, USA	10-year longitudinal cohort study	FFQ; Quartile 1: 597-1921 mg/day; Quartile 2: 1921-2511 mg/day; Quartile 3: 2517-3264 mg/day; Quartile 4: 3264-11565 mg/day	3MS at year 3 and year 10; Micro- and Macro-structural MRI indices for 248 participants at year 10.	Sodium intake was not associated with increased odds of incident cognitive impairment.
Sodium intake and physical activity impact cognitive maintenance in older adults: the NuAge Study	*N* = 1,262 Age: 67–84 Cognitively normal Quebec, Canada	5-year prospective longitudinal Study	FFQ; Low tertile 677-2263 mg/day, Mid tertile 2263-3090 mg/day, High tertile 3091-8098 mg/day	3MS administered at baseline and annually for 3 additional years	Low sodium tertile displayed better cognitive performance over time compared with the high and mid tertiles
Hypertension, Dietary Sodium, and Cognitive Decline: Results from the Women’s Health Initiative Memory Study	*N* = 6,426 Age: 65–79 Cognitively normal Women’s Health Initiative Clinical Centres, USA	Parallel randomized clinical trial, median follow-up of 9.11 years	FFQ, corrected via 24-h urine excretion analyses; sodium intakes: <1500 mg/day, 1501-2999 mg/day > 3000 mg/day	3MS baseline assessment, then repeated annually. Diagnosis of MCI or PD.	Sodium intake > 1,500 mg/day did not alter the risk for cognitive decline in hypertensive women or women with antihypertensive treatment. Sodium intake > 2,300 mg/day was associated with higher risk for cognitive decline in women with hypertension, whereas sodium intake < 2,300 mg/day did not significantly increase this risk. However, formal tests for interaction were not significant.
Lifestyle and neurocognition in older adults with cognitive impairment	*N* = 160 Age: >55 Cognitive impairment without dementia Duke Aging Center and Duke Alzheimer’s Disease Research, USA	2-by-2 factorial (exercise (AE)/no exercise and DASH diet/no DASH diet) 6 month randomized clinical trial	Sodium intakes: AE: 2,407-2,858 mg/day; DASH: 1,550-1,987 mg/day; AE+DASH: 1,640-2,095 mg/day; Control 2,175-2,661 mg/day	TMT, Stroop Test, Digit Span Forward and Backward, Digit Symbol Substitution Test, Ruff 2 & 7 Test, Animal Naming	DASH diet was not related to improved cognition, participants who engaged in both exercise and DASH diet compared to the Health Education control group had a greater benefit.
Genetic Factors of Alzheimer’s Disease Modulate How Diet is Associated with Long-term Cognitive Trajectories – A UK Biobank Study	*N* = 1,787 Age: 46–77 United Kingdom	10-year Prospective cohort study	FFQ	FIT at baseline and two follow up assessments (2006–2010, 2012–2013, 2015–2016)	Adding salt to food detrimental for those who have either a family history of AD or APOE ɛ4 allele.
Association Between Dietary Sodium Intake and Cognitive Function in Older Adults	*N* = 925 Mean age: 74.5 ± 8.7 Southern California, USA	Cross-sectional study	FFQ; Quartile 1 1647.5 ± 540.4; Quartile 2 1814.4 ± 602.3; Quartile 3 1950.5 ± 522.3; Quartile 4 2628.5 ± 824.9	Verbal Fluency Test, MMSE, and TMT	Lower sodium intake associated with increased odds of cognitive impairment including a longer time to complete TMT B and lower MMSE score.
The relationship between nutrient intake and cognitive performance in people at risk of dementia	*N* = 44 (32 women, 12 men) Mean age: 57.7 ± 9.4 Ireland	Cross-sectional study	FFQ	MMSE; Picture-Word Learning Test; Letter-Digit Coding Test; Stroop Colour-Word Test	Intakes of sodium and cholesterol were significantly higher in subjects with altered MMSE scores, indicating there is a link between cholesterol and sodium intake, and cognitive impairment among individuals at genetic risk of dementia.
Protein and thiamin intakes are not related to cognitive function in well-nourished community-living older adults	*N* = 47 (30 women, 17 men) Mean age: 78.2 ± 6.1 Independent-living facility residents in Illawarra, NSW, Australia	Cross-sectional study	Three-day food diary	Verbal Fluency Test; RAVLT; BNT; Digit-Span Backwards Test; TMT; Geriatric Depression Scale.	Men had higher sodium intake than women. No association between sodium intake and cognitive function.
Association between 24-h urinary sodium to potassium ratio and mild cognitive impairment in community-based general population	*N* = 561 Age ≥ 35 Emin, Northern Xinjiang, China	Cross-sectional study	24-h urine to determine sodium to potassium ratio; Tertile 1 ≤ 3.918; Tertile 2 3.919–5.905; Tertile 3 ≥ 5.906	MMSE	Higher urinary sodium to potassium ratios were associated with mild cognitive impairment.
Diet quality and cognitive function in mid-aged and older men and women	*N* = 617 Mean age: 60.2 ± 3.14 Victoria, Australia	Prospective, population based longitudinal cohort study	FFQ measuring salt added during or after cooking.	TICS-m	Men who reported ‘usually’ adding salt during cooking displayed poorer cognitive function than men who never added salt. Women who ‘sometimes’ added salt after cooking, performing better in cognitive tests than women who never added salt.
Excessive Dietary Salt Intake Exacerbates Cognitive Impairment Progression and Increases Dementia Risk in Older Adults	*N* = 2041 Age ≥ 60 Shandong Area, China	Prospective, population-based cohort study	Seven-days urinary sodium measurements.	MMSE, MoCA, DRS and IQCODE in Elderly. APOE genotype,	Global cognitive function decreased progressively faster with increasing of salt intake, independently of known risk factor (including hypertension and APOE genotype).
Independent and interactive associations of dietary nitrate and salt intake with blood pressure and cognitive function: a cross-sectional analysis in the InCHIANTI study.	*N* = 1271 Age > 50 Chianti region, Tuscany, Italy	Cross-sectional analysis	FFQ: 24-h sodium urinary excretion: Sodium intake: 2357.9 ± 848.9 mg/day Nitrate intake: 89.9 ± 73.3 mg/day	MMSE, TMT	Urinary sodium and dietary sodium intake were not associated with any cognitive performance in the study population. High nitrate levels were associated with lower BP values and results were not modified by sodium concentrations.
Association of dietary sodium, potassium, sodium/potassium, and salt with objective and subjective cognitive function among the elderly in China: A prospective cohort study	*N* = 4213 Age ≥ 50 11 provinces, 4 autonomous cities in China	Prospective cohort study	24/3 dietary recall Sodium intake: Quartile 1: 2986.8 mg/day: Quartile 2: 4258.7 mg/day: Quartile 3: 5593.2 mg/day: Quartile 4: 8067.8 mg/day	TICS-m: self-report of memory status and memory change	Lower sodium and sodium/potassium, and higher potassium intakes might benefit cognitive functions: this effect might be mediated by CCVD. The association between salt and cognitive function might be mediated by sleep time.

*Abbreviations: 3MS* Modified Mini-Mental State Examination, *AD* Alzheimer’s disease, *APOE* apolipoprotein E, *BNT* Boston Naming Test, *DRS* Dementia Rating Scale, *FFQ* Food Frequency Questionnaire, *FIT* Fluid Intelligence Test, *IQCODE* Informant Questionnaire on Cognitive Decline in the Elderly, *MoCA* Montreal Cognitive Assessment, *MMSE* Mini-Mental State Examination, *MRI* magnetic resonance imaging, *NSW* New South Wales, *PD* probable dementia, *RAVLT* Rey Auditory Verbal Learning Test, *SMMSE* Standardized Mini-Mental State Examination, *TICS-m* Telephone Interview for Cognitive Status Modified, *TMT* Trail Making Test

## Mechanisms Of Action

Currently, the potential mechanisms underlying the relationship between high sodium intake and poorer cognitive function are not completely understood, with the majority of proposed mechanisms being deduced from animal models. Higher 24-hour sodium excretion has been associated with higher serum C-reactive protein levels, a measure of systemic inflammation and a risk factor for both CVD and dementia, in 1597 human participants [36]. Notably, several rodent studies have demonstrated that a high salt diet is associated with greater oxidative stress, particularly in the hippocampal region, which leads to neuronal cell death [9, 10, 16]. The most abundant reactive oxygen species (ROS) formed during the course of cellular metabolism is the superoxide radical which is implicated in neuronal death in the hippocampus. A high salt diet increases superoxide formation by increasing nicotinamide adenine dinucleotide phosphate (NAPDH) oxidase activity [10], and this is accompanied by decreased antioxidant capacities (the antioxidant defence system that works to remove ROS). This suggests that the hippocampus suffers oxidative damage under a high salt diet, which contributes to cognitive dysfunction [9]. Furthermore, oxidative stress may cause the formation of impaired calcium ion-mediated signalling and reduced sodium–potassium adenosine triphosphatase (Na^+^/K^+^ATPase) enzyme expression, which leads to disturbed synaptic plasticity and cognitive impairment [37].

Another potential underlying mechanism that may explain the association between high dietary salt intake and poorer cognitive function is hypertension. In particular, Guo et al. [14] found that rats exposed to a high salt diet showed cognitive deficits accompanied by a significant increase in systolic blood pressure and cerebral hypoperfusion. Moreover, hypertension and cerebral hypoperfusion in high salt-treated rats was associated with reduced synaptogenesis and the loss of dendritic spines in the hippocampus, which plausibly explains the spatial memory deficits on testing. Additionally, the study found that high salt-treated rats exhibited decreased activity in the CaMKII/CREB signalling pathway, which is important in learning and memory. The study found that a high salt diet was not associated with pathological features of AD, with no differences in levels of Aβ-amyloid and tau phosphorylation found between rats exposed to high or normal salt diets [14]. These results were partially confirmed by Kubota et al. [18] using male C57BL/6 N mice: while data regarding the correlation between high salt-induced hypertension and impaired behaviours are strong, together with degenerated neuronal morphology data, the authors observed abnormal tau hyperphosphorylation after high salt intake. The study looked at the mechanism under the correlation between hypertension and cognitive impairment by using losartan (a non-BBB-crossing angiotensin receptor blocker): interestingly, an interaction between AngII-AT1 and PGE2-EP1 systems was found, suggesting a potential therapeutic target for hypertension-induced dementia. A recent study by Faraco et al. [17], however, showed that mice fed a high salt diet did exhibit cognitive impairment and increased hyperphosphorylation and aggregation of tau. Importantly, the study showed that high salt diet induced tau hyperphosphorylation was caused by a deficiency in endothelial nitric oxide and activation of cyclin dependant kinase 5. This was further supported by the finding that mice exposed to a high salt diet no longer exhibited cognitive impairment and tau hyperphosphorylation when endothelial nitric oxide was replenished. Moreover, the study showed that high salt diet-induced cognitive impairment was dependent on the hyperphosphorylation of tau and not due to cerebral hypoperfusion, as tau knockout and tau antibody-positive mice exposed to a high salt diet did not demonstrate cognitive impairment despite cerebral hypoperfusion.

While no studies to date have investigated the direct relationship between a high salt diet and Aβ-amyloid, a study found that a combination of a high salt and high cholesterol diet induced cognitive impairment and increased Aβ-amyloid peptide aggregation in rats [38]. In addition, in vitro studies have shown that cells (HEK293 cells (primary; human embryonic kidney cell) overexpressing amyloid precursor protein or C99 fragment) treated with sodium chloride have a higher aggregation of Aβ-amyloid than control cells, suggesting that high sodium impairs Aβ-amyloid clearance, likely via downregulation of APOE [39]. Very few studies, however, have examined the mechanisms underlying the relationship between a high salt diet and cognitive function in humans, with only one MRI study demonstrating that a high salt diet was not associated with structural brain changes [29]. While no published studies have investigated the relationship between high sodium intake and cerebral Aβ-amyloid, human studies have shown that hypertension (for which high sodium intake is a risk factor) is associated with greater Aβ-amyloid load [40] and may modulate the relationship between Aβ-amyloid and development of AD [41]. In particular, Shah et al. [41] showed that decreased Aβ-amyloid plasma levels were associated with increased risk of AD in 667 Japanese American males and that this risk increased with higher diastolic blood pressure. Importantly, the study showed that decreased plasma Aβ-amyloid was associated with increased cerebral amyloid angiopathy, consistent with decreased plasma Aβ-amyloid reflecting deposition and aggregation of Aβ-amyloid in the brain.

## Discussion

In summary, for the human studies we reviewed one randomised controlled trial (duration six months), six longitudinal observational studies ranging from 3 years to 11 years, and seven cross-sectional observational studies. Sodium intake was measured using FFQs, three-day food records, four-day food records, 24-hour urinary sodium, and 24-hour urinary sodium to potassium ratios. Of the 14 studies reviewed in the human studies section, 10 found associations with sodium intake in at least one cognitive domain (global cognition, executive function, fluid intelligence, and cognitive switching) or with MCI diagnosis. There were no common methodological approaches amongst the studies reporting effects compared to those reporting no effect, with a wide range of age groups included, varying sodium intake measurement methods and four studies incorporating the additional variables of physical activity, hypertension, and genetic risk factors. Overall, mixed results were found regarding whether dietary sodium intake is associated with cognitive function, making it difficult to draw a clear conclusion. However, the findings of this review partly support the notion that high sodium intake could be having a negative impact on middle and older-aged individual’s cognitive health, although this relationship needs further exploration.

While self-reported sodium intake can be informative and was the most commonly used method in the studies reviewed here, the addition of 24-hour urinary sodium levels would enhance research findings and its interpretation. The accuracy of self-reported dietary intake data can vary depending on one’s ability to recall dietary information. Differing length of FFQs, as well as differences in nutrient databases used for determining sodium levels, may have led to estimates of sodium intake differing between studies. Furthermore, some FFQs do not assess the addition of salt during cooking or at the table, leading to an underestimation of actual sodium intake. As shown in Haring et al. [12], correction of sodium estimates based on FFQ by use of 24-hour sodium excretion resulted in higher estimates of daily sodium intake, with McGrattan et al. [35] confirmed that dietary sodium intake assessed by FFQ was not associated with objective sodium assessment (24-hour urine excretion). These differing methods risk the under-reporting or over-reporting of sodium intake and could explain the lack of significant results in some studies and the mixed results observed overall.

A longer follow-up time may be required to truly capture the effects of sodium intake on cognitive change and risk of dementia in late life. These longitudinal studies minimise the potential influence of residual confounding of associations observed. Observational studies, as the majority of the human studies currently published are, cannot infer causality. Individuals could have restricted their sodium intake due to adverse health conditions that could have also impacted their cognitive performance. Furthermore, multiple measurements of sodium intake would improve analysis by allowing for the assessment of change in these baseline factors over the observational period, which may better explain study findings. Collectively, data reported in this review indicate that further exploration of the relationship between dietary sodium intake and cognition is needed in well characterised human cohorts, using comprehensive assessment of cognitive function.

## Future Research

While animal studies demonstrate a consistent association between high sodium intake and cognitive impairment, this relationship needs further evaluation in humans. Existing studies are limited in that they have primarily explored global cognition using measures with limited sensitivity. Overall, additional prospective studies conducted in diverse populations, and adequately powered intervention studies with long durations, are required to thoroughly examine the effect of sodium intake in humans on clinically relevant cognitive and cerebral outcomes. Moreover, the development of maximum recommended sodium intake levels remains to be fully determined. Whilst animal models have shown promising results with interventions of timeframes such as two months, this represents a considerably larger percentage of total lifespan for animals than it does for humans, and a longer timeframe could be needed to show enhanced outcomes in human trials. Both acute and chronic effects of sodium intake also need to be investigated using neuroimaging techniques in conjunction with cognitive and physiological measures to further elucidate the underlying biological mechanisms. Furthermore, it should be acknowledged that studies in cognitively normal adults are unlikely to demonstrate large improvements in cognitive function, and therefore it is imperative that suitable, sensitive cognitive tests are utilised. There is currently no clear evidence regarding the specific domains of cognition that sodium intake impacts, with future studies requiring a range of cognitive domains to be investigated in order to determine those cognitive domains most likely to benefit from a lower sodium diet. Future studies should also include robust blood and cerebral biomarkers of AD, including inflammatory marker levels in the blood, cerebrospinal fluid biomarkers, Aβ-amyloid levels, and cerebral volumes, known to correlate with pathological progression.

If emerging evidence continues to suggest significant cognitive deficits associated with sodium intake, another important consideration is the optimum age for the recommendation of lowering sodium intake. Indeed, the neuropathological hallmarks of AD begin to accumulate 15–20 years before symptoms manifest, implying that the optimal stage of life could be middle-aged or younger.

## Conclusions

As populations continue to focus on developing strategies to promote healthy ageing, dietary interventions involving decreasing sodium intake represent a promising avenue for future research. However, many questions still need to be answered before a definite conclusion can be made regarding the extent to which lower consumption of sodium can protect the ageing brain and lowering intake can be included in public health dietary recommendations.

## Supplementary Information

Below is the link to the electronic supplementary material.

Supplementary File 1 (DOCX 14.8 KB)

## Data Availability

No datasets were generated or analysed during the current study.
