# Supplementary material for: The Relationship Between Dietary Sodium Intake and Cognitive Function: A Narrative Review
Source: Curr Nutr Rep. 2026 Mar 21;15(1):29. doi: 10.1007/s13668-026-00750-8 (PMC13004737; doi:10.1007/s13668-026-00750-8)
Supplement: Supplementary file 1 — (DOCX 14.8 KB) [file 13668_2026_750_MOESM1_ESM.docx]

**Supplementary Table 1:** Search terms for finding manuscript articles

|  | **Search terms** |
| --- | --- |
| **Human studies** | ("sodium"[tiab] OR "salt"[tiab]) AND ("executive function"[tiab] OR "language"[tiab] OR "verbal fluency"[tiab] OR "memory"[tiab] OR "cognitive decline"[tiab] OR "subjective memory complaints"[tiab] OR "mild cognitive impairment"[tiab] OR "Alzheimer's disease"[MH] OR "dementia"[tiab] OR "cognition"[tiab] OR "cognitive performance"[tiab] OR "global cognition"[tiab] OR "cognitive impairment"[tiab] OR "cognitive composites"[tiab] OR "cognitive function"[tiab] OR "fluid intelligence"[tiab] OR "working memory"[tiab] OR "learning"[tiab] OR "processing speed"[tiab] OR "neuropsychological tests"[tiab] OR "Montreal Cognitive Assessment"[tiab] OR "MoCA"[tiab] OR "MMSE"[tiab] OR "Mini-Mental State Examination"[tiab] OR "MRI"[tiab] OR "magnetic resonance imaging"[tiab] OR "fMRI"[tiab] OR "PET"[tiab] OR "brain volume"[tiab] OR "hippocampal atrophy"[tiab] OR "amyloid"[tiab] OR "tau"[tiab] OR "biomarkers"[tiab]) AND ("humans"[MH] OR "adult"[tiab] OR "elderly"[tiab] OR "older adults"[tiab] OR "healthy subjects"[tiab] OR "clinical population"[tiab]) |
| **Animal studies** | ("sodium"[tiab] OR "salt"[tiab]) AND ("spatial learning"[tiab] OR "spatial memory"[tiab] OR "memory"[tiab] OR "object-place recognition"[tiab] OR "fear conditioning"[tiab] OR "short-term memory"[tiab] OR "object recognition"[tiab] OR "active avoidance"[tiab] OR "cognition"[tiab] OR "cognitive function"[tiab] OR "cognitive decline"[tiab] OR "cognitive performance"[tiab] OR "cognitive impairment"[tiab] OR "learning"[tiab] OR "working memory"[tiab] OR "executive function"[tiab] OR "recognition memory"[tiab] OR "decision making"[tiab] OR "attention"[tiab] OR "behaviour"[tiab] OR "behaviour task"[tiab] OR "Morris water maze"[tiab] OR "Barnes maze"[tiab] OR "radial arm maze"[tiab] OR "MRI"[tiab] OR "magnetic resonance imaging"[tiab] OR "fMRI"[tiab] OR "PET"[tiab] OR "brain volume"[tiab] OR "hippocampal atrophy"[tiab] OR "amyloid"[tiab] OR "tau"[tiab] OR "biomarkers"[tiab]) AND ("rats"[tiab] OR "mice"[tiab] OR "rodents"[tiab] OR "primates"[tiab] OR "non-human primates"[tiab] OR "animal model"[tiab] OR "experimental animals"[tiab]) |

Abbreviations: tiab, title, abstract, and keywords (or collection title and other abstract); MH, Medical Subject Headings.
